# A novel instrument of cognitive and social congruence within peer-assisted learning in medical training: construction of a questionnaire by factor analyses

**DOI:** 10.1186/s12909-020-02129-x

**Published:** 2020-07-08

**Authors:** Teresa Loda, Rebecca Erschens, Christoph Nikendei, Katrin Giel, Florian Junne, Stephan Zipfel, Anne Herrmann-Werner

**Affiliations:** 1 Department of Internal Medicine VI, Psychosomatic Medicine and Psychotherapy, University Hospital Tuebingen, Osianderstr. 5, D-72076 Tuebingen, Germany; 2 Centre for Psychosocial Medicine, Department of General Internal Medicine and Psychosomatics, University Hospital Heidelberg, Heidelberg, Germany; 3 Deanery of Students’ Affairs, University’s Faculty of Medicine, Tuebingen, Germany

**Keywords:** Cognitive and social congruence, Factor analysis, Medical education, Peer-assisted learning

## Abstract

**Background:**

Peer-assisted learning is effective due to cognitive and social congruence. Cognitive congruence is created by sharing a similar knowledge base between students and student tutors. Social congruence is defined as having similar social roles. A questionnaire of these concepts was newly constructed, and this study explored the factor analysis of the instrument.

**Methods:**

In a cross-sectional method design cognitive and social congruence were operationalised by exploratory and confirmatory factor analyses. Cognitive and social congruence were assessed by validated questionnaires and through self-developed items that were collected through semi-structured interviews.. The questionnaire consisted of 26 items that were rated on a five-point Likert scale, from 0 = I strongly disagree to 4 = I strongly agree.

**Results:**

676 medical students participated in the study. Exploratory factor analysis for students resulted in a two-factor solution with cognitive and social congruence as confirming factors. New findings showed that the items “non-judgmental learning atmosphere” and “informal communication” were associated to cognitive congruence, “effectiveness” and “comprehensible explanations” belonged to social congruence. Confirmatory factor analysis for student tutors confirmed the resulting two-factor solution.

**Conclusions:**

As one of the largest investigation of cognitive and social congruence, this study investigated the underlying mechanisms of effective PAL using factor analysis. Cognitive congruence was created by sharing the same knowledge. Knowledge transfer might play a relevant role in cognitive congruence. Social congruence focused on the relationship between student tutors and students, which might impact the content level. Practical recommended actions (using the same language) could be implemented.

## Background

The concept of peer-assisted learning (PAL) presents a well-established methodological ingredient in the medical curriculum [1–3]. PAL is based on the idea that peers mostly in their upper years of study in medical school teach students. Past studies determined that PAL is effective especially because of cognitive and social congruence between student tutors and students [2, 4–7].

Cognitive congruence is created by a common and similar knowledge base between student tutors and students [6, 7]. As a result, the student tutor may explain difficult topics at a level students can comprehend using a familiar language [7–9]. Social congruence, on the other hand, is defined as the student tutor and their students sharing similar social roles such as being medical students [6, 7]. Student tutors show social congruence by being interested in the students’ problems and demands [10, 11]. Moreover, students are motivated by the fact that their student tutors have successfully completed the course and passed the associated exam [6].

Cognitive and social congruence between students and student tutors fosters a relaxed and pleasant learning environment and may result in a powerful peer-assisted learning experience [1, 3, 6, 7, 12, 13]. Student tutors perceived as cognitively and socially congruent by students are considered empathic and supportive by sharing learning experiences and giving alternative proposed solutions [6, 7, 14]. Students, in turn, are encouraged by socially and cognitively congruent student tutors to actively participate in class, ask questions and give feedback [3, 15, 16]. Finally, cognitive and social congruence may also contribute to increasing students’ motivation to study [17].

Despite the increasing relevance of cognitive and social congruence in peer learning addressed by many studies, these two concepts are not often studied. Previous studies investigated the effectiveness of peer teaching or tutorials led by student tutors and consequently assumed that cognitive and social congruence might be the reason for an efficient class [18–21]. Hence, cognitive and social congruence might be seen as kind of a “black box”, as there is no practical evidence of both constructs at a behavioural level [18]. Vygotsky postulated that humans generate knowledge and meaning from the interaction between experiences and ideas and therefore construct their own knowledge which might represent cognitive congruence [22]. Further, there is no empirical evidence of how student tutors and students could become socially and cognitively congruent.

In the literature, we found several studies that assessed cognitive and social congruence [2, 7, 23]. Schmidt & Moust [7] developed items to assess cognitive and social congruence as well as expertise. This questionnaire was found to be valid and reliable [24]. However, literature showed that there are various ways to measure cognitive and social congruence [2, 6, 11, 16, 25–27]. In this context, the practical and behavioural part of cognitive and social congruence were neglected [18]. The questionnaire of Vaughan & Macfarlane [27], e.g., did not explicitly assess cognitive and social congruence, but the behaviour of student tutors and students that might reflect both concepts. Prior findings were used to develop a questionnaire that should assess cognitive and social congruence in their full dimension with focus on behavioural aspects of student tutors and students that strengthen cognitive and social congruence.

## Methods

### Aim

This study aimed to operationalise cognitive and social congruence, on a behavioural level of student tutors and students by using a newly developed questionnaire. The perspectives of students as participants in tutorials and of student tutors were measured to comprehensively cover the full dimension (students’ and student tutors’ perceptions) of cognitive and social congruence in tutorials, including behaviour. The constructed questionnaire of cognitive and social congruence was tested by exploratory and confirmatory factor analysis separated for student and student tutors.

### Ethics

The study received ethical approval from the Ethics Committee of Tuebingen Medical Faculty (No. 129/2017BO2) in April 2017. The participation was on voluntary base and all medical students and student tutors provided their written informed consent.

### Design, participants and procedure

This study presents the first operationalisation of cognitive and social congruence with focus on the behavioural level of students and student tutors in a cross-sectional design with a quantitative questionnaire survey. Medical students and student tutors from the Medical Faculty of Tuebingen were invited to participate. The medical students were from different years of study, ranging from first until the final year of study (for details, see results). Student tutors came from various fields such as medical history, anatomy, internal medicine, physiology, skills lab, and surgery. They were recruited from different classes within their usual mandatory courses. As reimbursement, books and vouchers were raffled among the participants.

### Measurements

Demographic information such as gender, age, year of study, and questions belonging to tutorials (e.g. subject of the tutorial) were included in the questionnaire. Additionally, student tutors were asked about their qualifications, their number of lessons which they have already taught and their discipline as student tutors in both measurements.

### Description of the survey

The survey was based on the instruments used by Schmidt & Moust [7] and Vaughan & Macfarlane [27]. All items of both questionnaires were translated into German. Seven further items were developed based on previous literature review (see also [6, 11, 28]) and semi-structured interviews on cognitive and social congruence that were conducted and analysed (Loda, T, Erschens, R, Nikendei C, Zipfel S, Herrmann-Werner A: Qualitative analysis of cognitive and social congruence in peer-assisted learning – the perspectives of medical students, student tutors and lecturers, submitted).

#### Final survey instrument

The final questionnaire, hence, consisted of 26 items. Eight of 26 items were meant to be associated with cognitive congruence, and 15 of 26 items were expected to belong to social congruence. Two items assessed the student tutors’ expertise, and one item evaluated the tutorials in general. The questionnaire was conducted in both an online and a paper- version to apply to students from all semesters. The survey responses were anonymous. Students and student tutors indicated their level of agreement with the items using a five-point Likert scale, from 0 = I strongly disagree to 4 = I strongly agree. We used the same Likert scale as Schmidt & Moust [7] and Vaughan & Macfarlane [27] but changed the endpoints from 1 to 0 and 5 to 4. Social congruence was measured with items such as “The tutor proved to be interested in me as student and learner” or “There was a supporting and trustful learning basis between tutor and students”. Measures of cognitive congruence were] similar to questions such as “The tutor was able to explain issues adapted to the students’ language and knowledge” or “The tutor asked students questions they were well able to understand”. The questionnaire was separated into students’ and student tutors’ view. Students could only answer the students’ perspective. Student tutors could fill the students’ as well as the student tutors’ perspective.

### Statistical analysis

The questionnaire was evaluated quantitatively using IBM SPSS Statistics version 25 and Amos 25. Mean values, standard deviations, frequencies, and percentages of relevant factors were calculated. Missing data were replaced by parameters using the full information maximum likelihood method. The overall mean of missing values was estimated as 1.28%. Missing values were considered only if at least 80% of each of the questionnaires had been completed.

To assign the items to factors of cognitive and social congruence, exploratory factor analysis for students’ sample and confirmatory factor analysis for student tutors’ sample were conducted, respectively. For the exploratory factor analysis the principal component analysis was used as method of extraction and varimax rotation was determined as method of rotation. The number of factors of the exploratory factor analysis were determined to be two based on the Kaiser Criterion, also called the Eigenvalue Rule [29, 30] and scree plot [31]. Previously, the correlation structure was checked by regarding the correlation matrix and its inverse one. Further, we calculated the Kaiser-Meyer-Olkin (KMO-) value and conducted the Bartlett’s test of sphericity [32–34]. The prerequisites for the factor analysis were examined and given (please see results for details).

To interpret the factors, rotated factor loading was used. Factors were interpreted if at least four variables had a load of ±0.6 or higher or if at least 10 variables had a load of ±0.4 or higher. Item 6 (“Tutors asked understandable questions”) and item 23 (“Tutor and I share similar roles”) were eliminated because of their loading less than ±0.2. The reliability was tested using Cronbach’s alpha coefficient.

**Fig. 1:**
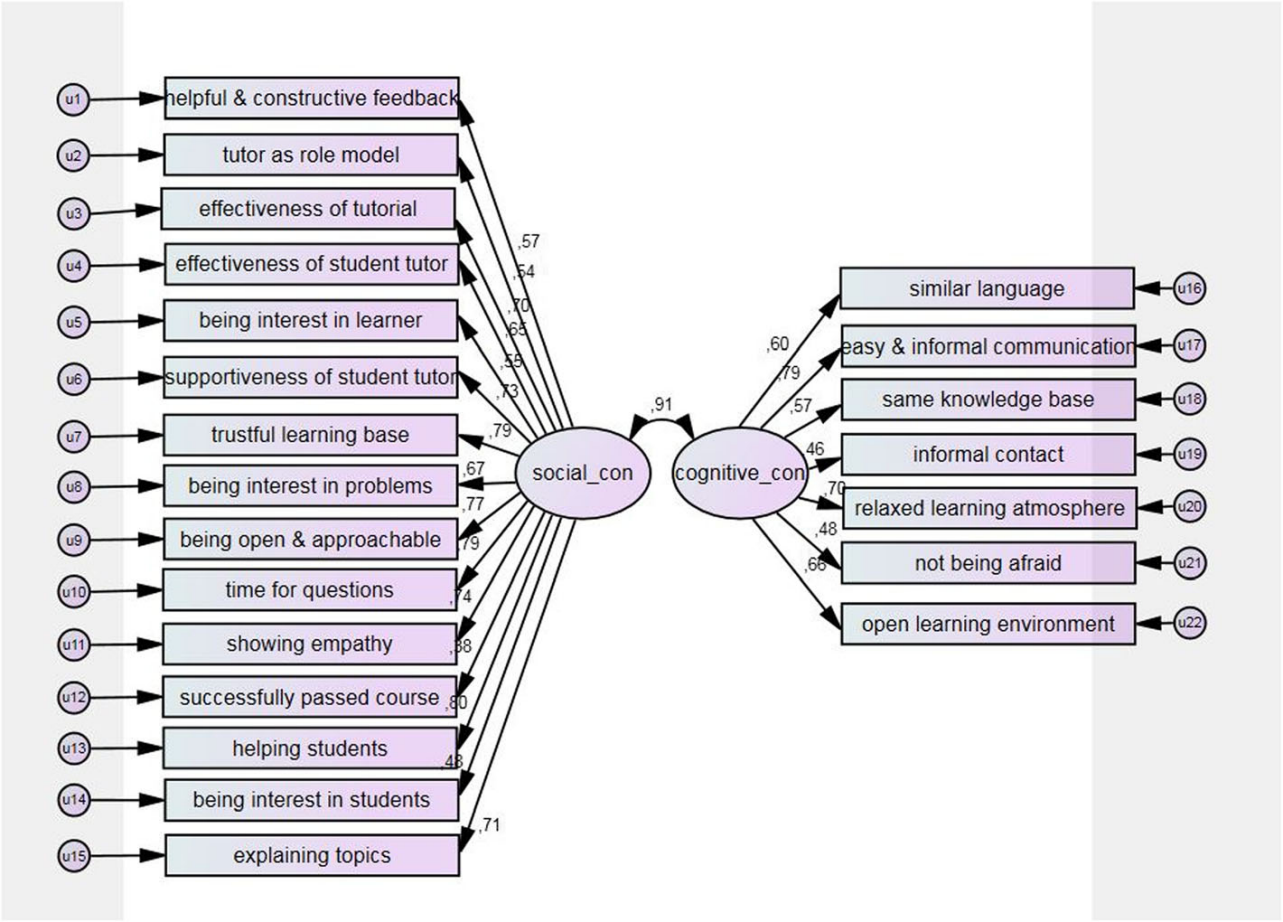
Confirmatory factor analysis of cognitive and social congruence. The graphic shows the correlation of cognitive or social congruence with the various items and their residuals. Social and cognitive congruence are highly correlated with r = 0.91. Item 5, e.g., presents being interested in students’ problems. Item 12, e.g., presents similar language

To examine the resulting factor model based on exploratory factor analysis, a confirmatory factor analysis with student tutors’ data was conducted. There was no significant difference between the students and student tutors. Robust maximum likelihood was used for parameter estimation. The model specification can be seen in Fig. 1. For the evaluation of the model fit, the following fit indices were applied: Chi-Square test, the quotient of Chi-Square and degrees of freedom, Root Mean Square Error of Approximation (RMSEA), and Comparative Fit Index (CFI). For a good model fit, the quotient of Chi-Square and degrees of freedom was ≤2.5 [35]. Regarding the RMSEA, a value of ≤0.05 was interpreted as a close fit; a value of ≤0.08 was interpreted as an acceptable fit [36]. For CFI, a cut value of ≥0.9 was applied as acceptable and ≥ 0.95 as good fit [37, 38].

## Results

### Samples

Six hundred seventy-six (RR = 79.5%) medical students, where 22.1% taught as student tutors (RR = 60.8%), filled in the questionnaire; 59.4% were female, and the average age was *M* = 24.17 ± 3.84. The medical students came from different years of study (15.3% = 1st year of study, 26.9% = 2nd year of study, 26.0% = 3rd year of study, 14.9% = 4th year of study, 11.6% = 5th year of study, 5.0% = final year). As to be expected, student tutors came, on average, from more advanced years of study (0.7% = 1st year of study, 6.2% = 2nd year of study, 28.7% = 3rd year of study, 24.0% = 4th year of study, 34.3% = 5th year of study, 6.2% = practical year). Please see Table 1 for single items.

**Table 1:** Descriptive data of the single items

Items	Medical Students		Student Tutors	
	Mean	SD	Mean	SD
I1 Same knowledge base	2.60	0.88	2.30	0.90
I2 Similar language	3.34	0.75	3.21	0.92
I3 Preferring informal contact	2.83	0.92	3.21	0.86
I4 Student wasn’t afraid to tell tutor if they didn’t understand anything	3.22	0.88	2.95	1.03
I5 Being interested in students’ needs and problems	2.99	0.85	3.40	0.65
I7 Helping students	3.20	0.76	3.34	0.76
I8 Tutor was able to explain students the topics based on their language and knowledge base	3.25	1.51	3.34	0.74
I9 Taking time for questions	3.20	0.79	3.62	0.69
I10 Supportiveness of student tutor	3.08	0.82	3.44	0.76
I11 Showing empathy	2.88	0.82	3.30	0.74
I12 Being interested in student as learner	2.72	0.84	3.14	0.73
I13 Effectiveness of the student tutor	2.52	0.89	2.80	0.76
I14 Being open and approachable	3.21	0.75	3.53	0.67
I15 Helpful and constructive feedback	2.34	0.92	2.82	1.01
I18 Seeing tutor as role model	2.14	1.06	2.55	0.96
I19 Stress-free and relaxing learning atmosphere	2.80	1.00	3.01	0.96
I20 Being interested in students	2.21	1.04	2.69	0.94
I21 Easy and informal communication	3.11	0.83	3.43	0.73
I22 Trustful learning base	2.81	0.82	3.24	0.76
I23 Creating an open and non-judgmental learning environment	2.90	0.82	3.30	0.75
I24 Successfully passed the course	2.55	1.26	2.85	1.08
I26 Effectiveness of tutorial	3.12	0.87	3.42	0.82

### Prerequisites for exploratory factor analysis

The correlation structure was suitable because the inverse correlation matrix represented a diagonal one with values outside the diagonal close to zero while the values of the diagonal are higher. In order to reduce data the principal component analysis (PCA) was used as method of extraction and varimax rotation was determined as method of rotation. The KMO value was marvellous with 0.96 [32, 33]. Additionally, the Bartlett’s test of sphericity [34] showed that the variables were not completely uncorrelated (χ^2^ (231, *N* = 527) = 5397.75, *p* < .001;). The number of factors of the exploratory factor analysis were determined to be two based on the Kaiser Criterion, also called the Eigenvalue Rule [29, 30] and scree plot [31]. For students, the scree plot and the Kaiser Criterion indicated a two-factor solution with Eigenvalue (1) = 9.92, Eigenvalue (2) = 1.32 and an overall variance of 51.07%. Please see appendix for more detailed results.

### Exploratory factor analysis: students

The exploratory factor analysis for students resulted in a two-factor solution. Based on the factor loadings, the results indicated that this two-factor solution could be assigned to cognitive (Factor 2) and social congruence (Factor 1). Cognitive congruence consisted of items that focused knowledge and learning environment with factor loadings from 0.513–0.766. The items of social congruence described the relationship and communication among students and student tutors with factor loadings from 0.401–0.693. Please see Table 2 for further details

**Table 2:** Factor loadings of cognitive and social congruence

Exploratory factor analysis				
Variable	λ (factor 1)	λ (factor 2)	Factor	
			1	2
I 15 Helpful and constructive feedback	0.693	0.120	+	
I 18 Seeing tutor as role model	0.690	0.190	+	
I 26 Effectiveness of tutorial	0.686	0.354	+	
I 13 Effectiveness of the student tutor	0.683	0.223	+	
I 12 Being interested in student as learner	0.682	0.364	+	
I 5 Being interested in students’ needs and problems	0.670	0.396	+	
I 10 Supportiveness of student tutor	0.677	0.472	+	
I 22 Trustful learning base	0.672	0.329	+	
I 14 Being open and approachable	0.661	0.400	+	
I 9 Taking time for questions	0.640	0.381	+	
I 11 Showing empathy	0.637	0.371	+	
I 24 Successfully passed the course	0.621	0.066	+	
I 7 Helping students	0.597	0.402	+	+
I 20 Being interested in students	0.527	0.380	+	+
I 8 Tutor was able to explain students the topics based on their language and knowledge base	0.401	0.072	+	
I 2 Similar language	0.175	0.766		+
I 21 Easy and informal communication	0.423	0.673		+
I 1 Same knowledge base	0.101	0.656		+
I 3 Preferring informal contact	0.139	0.634		+
I 19 Stress-free and relaxing learning atmosphere	0.433	0.575	+	+
I 23 Creating an open and non-judgmental learning environment	0.254	0.559		+
I 4 Student wasn’t afraid to tell tutor if they didn’t understand anything	0.461	0.513	+	+

Variables present the single items, λ = factor loadings and + shows which item loaded on factor 1 or 2 (cut-off: > .04). The loadings of the single items on factor 1 and factor 2 are depicted separately. Item 1, Item 2, Item 10, Item 11, Item 17-Item 24 are self-developed based on literature. PCA as method of extraction and varimax rotation

### Prerequisites for confirmatory factor analysis

The same assumptions as for the exploratory factor analysis were examined like the correlation structure. The KMO value was also marvellous with 0.92 [32, 33]. The Bartlett’s test of sphericity showed not completely uncorrelated variables with χ^2^ (276, *N* = 149) = 1346.92, *p* < .001. The results of the Kaiser Criterion and screeplot strengthened the two-factor solution with Eigenvalue (1) = 10.80, Eigenvalue (2) = 1.42 and an overall variance of 50.91%. Please see appendix for more detailed results.

### Confirmatory factor analysis: student tutors

After the evaluation of the students, we examined the resulting structure of the single items as a two-factor solution by conducting a confirmatory factor analysis with the student tutors’ data. We considered the student tutors’ data as acceptable, as they answered the same items but from their professional perspective.

The results showed the following fit indices of a two-factor solution with:

χ^2^ (208, *N* = 149) = 298.29, *p* < .001; χ^2^/df = 1.434 (≤2.5); RMSEA = 0.054 (90 % CI: 0.040; 0.067; ≤ 0.06) and CFI = 0.924 (≥0.90).

Although the chi-square test was significant, the other fit indices indicated an adequate model fit. As shown in Fig. 1, factor loadings of the single items in relation to cognitive and social congruence were moderate to high, ranging from 0.38 to 0.80. Further information of the factor loading can be seen in Fig. 1.

#### Cronbach’s alpha coefficient

The distribution of the items to cognitive and social congruence was found to be reliable, as Cronbach’s Alpha of cognitive congruence was 0.817 for students and 0.842 for student tutors, and Cronbach’s Alpha of social congruence was 0.913 for students and 0.927 for student tutors.

## Discussion

The aim of this study was to examine cognitive and social congruence in their full dimensions, including students’ and student tutors’ behaviour in tutorials, we tested this newly constructed questionnaire on cognitive and social congruence for German-speaking countries. The results of the exploratory factor analysis presented a two-factor solution regarding medical students including factor one as social congruence and factor two as cognitive congruence. The student tutors’ data confirmed this two-factor solution when conducting a confirmatory factor analysis.

### Main findings in light of previous evidence

The items in factor one might be related to social congruence, as it represented the social relationship between students and student tutors [7, 11]. Factor two could present cognitive congruence, as the loaded factors consisted of the intellectual and professional connection between students and student tutors [3, 4, 6–8]. When focussing on the two analyses, cognitive and social congruence were based on the concrete behaviour patterns of the student tutor and concrete aspects of the learning environment.

Our findings are mainly in line with previous research. Many studies postulated that cognitive congruence was created by sharing the same knowledge and using similar language among students and student tutors [3, 4, 6–9, 20, 39, 40]. Furthermore, cognitive congruence might contribute to a less stressful and relaxed learning atmosphere [1, 13, 41]. Social congruence could encourage student tutors to give feedback, take time for questions and react emphatically [3, 15, 16, 20, 42]. The studies of Moust & Schmidt [43] and Yew & Yong [11] postulated that student tutors showed social congruence by being more interested in students and their daily life, including needs and problems. Furthermore, social congruence contributed to a trusting and supportive learning relationship where student tutors behaved openly and in an approachable way [25, 26, 44, 45].

### Novel findings regarding cognitive and social congruence

In contrast to previous studies, an open and non-judgemental learning atmosphere was more strongly associated to cognitive congruence and not only to social congruence [6, 7, 11, 45]. Further, easy and informal communication as well as preferring informal contact with the student tutor was associated with cognitive congruence instead of social congruence as reported in the literature [6, 7, 11, 45]. This result could be explained by informal communication being associated with the way to best impart knowledge in tutorials. Thus, informal communication as part of knowledge transfer in tutorials belonged to cognitive congruence.

Although most studies reported that peer teaching was effective due to cognitive and social congruence [2, 17, 46, 47], our results showed that the effectiveness of the tutorial as well as of the student tutor might be associated with social congruence alone. This result is strengthened by the fact that the student tutors’ explanation based on the students’ language and knowledge base was also related to social congruence. As stated in various communication models, the social level seems to impact the content level of tutorials and seems to be crucial for the effectiveness of student tutor and tutorial [48, 49].

### Strengths and limitations of the study

This study could show several items in line with the literature [6, 7, 11] as well as new, interesting discoveries. This study presents the first operationalisation of cognitive and social congruence on a behavioural level of students and student tutors among German medical students. Furthermore, the student tutors’ perspective of cognitive and social congruence is, firstly, assessed. As the sample size among study participants was large and consisted of various semesters of medical education this study could be representative. However, when interpreting the results, one should remember possible limitations. We assumed an adequate model fit of student tutors’ data, though the chi-quadrat test was significant, and we retained the null hypothesis. The significant chi-quadrat test could be explained by the large sample size including 527 medical students and 149 student tutors [50]. Further, several items such as helping students were associated to both cognitive and social congruence. These items might explain the high correlation among cognitive and social congruence. Thus, the question raises if cognitive and social congruence can be regarded separately.

### Implications for teaching

For teaching implications, training courses focusing on relevant behavioural aspects of cognitive and social congruence resulting from this study could be developed and implemented for student tutors. Here, student tutors could specifically practice how to interact with the students as participants to be cognitively and socially congruent. Especially regarding the increasing heterogeneity among students due to future planning of interprofessional lectures, knowledge of cognitive and social congruence at a behavioural level becomes more relevant and should be a major focus in future research of peer tutoring.

## Conclusion

This study aimed to operationalise cognitive and social congruence of students and student tutors on a behavioural level. Cognitive congruence focused on teaching in the tutorials, including similar language and shared knowledge [7]. Social congruence represented the relationship between students and student tutors such as the student tutors’ general interest in the students [7, 11]. In contrast to previous studies, non-judgmental learning atmosphere and informal communication were associated with cognitive congruence instead of social congruence [6, 7, 11, 45]. Consequently, the way to best impart knowledge might result from cognitive congruence that should be also examined in future studies. Furthermore, student tutors’ explanation of topics of students’ language and knowledge base was related to social congruence [6–9, 12, 20]. Future studies should investigate if the social level might affect the content level in tutorials as reported by various theories of communication models and might impact the effectiveness of PAL [6, 48, 49].

## Supplementary information

**Additional file 1: Table 3.** Correlation matrix of exploratory factor analysis; all p (1-tailed) < .05. The correlation structure of the exploratory factor analysis was suitable because the inverse correlation matrix represented a diagonal one with values outside the diagonal close to zero while the values of the diagonal are higher. All *p*-values (1-tailed) were significant with < .05. **Table 4.** Correlation matrix of confirmatory factor analysis; all p (1-tailed) < .05. The correlation structure of the confirmatory factor analysis was suitable because the inverse correlation matrix represented a diagonal one with values outside the diagonal close to zero while the values of the diagonal are higher. All *p*-values (1-tailed) were significant with < .05. **Table 5.** Total variance explained of exploratory factor analysis. The total variance explained of exploratory factor analysis indicated a two component solution with 51.07% variance. The extraction method based on the Principal Component Analysis. **Table 6.** Total variance explained of confirmatory factor analysis. The total variance explained of confirmatory factor analysis presented a two component solution with 50.91% variance. The extraction method based on the Principal Component Analysis.

**Additional file 2: Figure 2.** Screeplot exploratory and confirmatory factor analysis. Both screeplots presented a two-factor solution: For students (exploratory factor analysis), the scree plot and the Kaiser Criterion indicated a two-factor solution with Eigenvalue (1) = 9.92 and Eigenvalue (2) = 1.32. For student tutors (confirmatory factor analysis), the results of the Kaiser Criterion and screeplot strengthened the two-factor solution with Eigenvalue (1) = 10.80 and Eigenvalue (2) = 1.42.

## Data Availability

The datasets used and/or analysed during this study are available from the corresponding author on reasonable request.

## Abbreviations

CI: Confidence interval
CFI: Comparative fit index
Df: Degrees of freedom
KMO: Kaiser-Meyer-Olkin
RMSEA: Root mean square error of approximation
I: Item
PAL: Peer-assisted learning
PCA: Principal component analysis
