# Supplementary material for: A novel instrument of cognitive and social congruence within peer-assisted learning in medical training: construction of a questionnaire by factor analyses
Source: BMC Med Educ. 2020 Jul 8;20:214. doi: 10.1186/s12909-020-02129-x (PMC7346370; doi:10.1186/s12909-020-02129-x)
Supplement: Supplementary file 1 — Additional file 1: Table 3. Correlation matrix of exploratory factor analysis; all p (1-tailed) < .05. The correlation structure of the exploratory factor analysis was suitable because the inverse correlation matrix represented a diagonal one with values outside the diagonal close to zero while the values of the diagonal are higher. All p-values (1-tailed) were significant with < .05. Table 4. Correlation matrix of confirmatory factor analysis; all p (1-tailed) < .05. The correlation structure of the confirmatory factor analysis was suitable because the inverse correlation matrix represented a diagonal one with values outside the diagonal close to zero while the values of the diagonal are higher. All p-values (1-tailed) were significant with < .05. Table 5. Total variance explained of exploratory factor analysis. The total variance explained of exploratory factor analysis indicated a two component solution with 51.07% variance. The extraction method based on the Principal Component Analysis. Table 6. Total variance explained of confirmatory factor analysis. The total variance explained of confirmatory factor analysis presented a two component solution with 50.91% variance. The extraction method based on the Principal Component Analysis. [file 12909_2020_2129_MOESM1_ESM.docx]

| Numbers of item | I1 | I2 | I3 | I4 | I5 | I7 | I8 | I9 | I10 | I11 | I12 | I13 | I14 | I15 | I18 | I19 | I20 | I21 | I22 | I23 | I24 | I26 |
| --- | --- | --- | --- | --- | --- | --- | --- | --- | --- | --- | --- | --- | --- | --- | --- | --- | --- | --- | --- | --- | --- | --- |
| I1 | 1.000 |  |  |  |  |  |  |  |  |  |  |  |  |  |  |  |  |  |  |  |  |  |
| I2 | 0.509 | 1.000 |  |  |  |  |  |  |  |  |  |  |  |  |  |  |  |  |  |  |  |  |
| I3 | 0.327 | 0.393 | 1.000 |  |  |  |  |  |  |  |  |  |  |  |  |  |  |  |  |  |  |  |
| I4 | 0.247 | 0.386 | 0.282 | 1.000 |  |  |  |  |  |  |  |  |  |  |  |  |  |  |  |  |  |  |
| I5 | 0.245 | 0.356 | 0.326 | 0.406 | 1.000 |  |  |  |  |  |  |  |  |  |  |  |  |  |  |  |  |  |
| I7 | 0.326 | 0.449 | 0.309 | 0.394 | 0.547 | 1.000 |  |  |  |  |  |  |  |  |  |  |  |  |  |  |  |  |
| I8 | 0.156 | 0.194 | 0.106 | 0.161 | 0.268 | 0.269 | 1.000 |  |  |  |  |  |  |  |  |  |  |  |  |  |  |  |
| I9 | 0.327 | 0.340 | 0.300 | 0.376 | 0.600 | 0.533 | 0.240 | 1.000 |  |  |  |  |  |  |  |  |  |  |  |  |  |  |
| I10 | 0.310 | 0.425 | 0.345 | 0.375 | 0.596 | 0.619 | 0.287 | 0.673 | 1.000 |  |  |  |  |  |  |  |  |  |  |  |  |  |
| I11 | 0.259 | 0.365 | 0.331 | 0.418 | 0.569 | 0.471 | 0.244 | 0.507 | 0.558 | 1.000 |  |  |  |  |  |  |  |  |  |  |  |  |
| I12 | 0.328 | 0.373 | 0.355 | 0.389 | 0.584 | 0.494 | 0.247 | 0.541 | 0.574 | 0.633 | 1.000 |  |  |  |  |  |  |  |  |  |  |  |
| I13 | 0.307 | 0.297 | 0.283 | 0.249 | 0.443 | 0.456 | 0.238 | 0.450 | 0.483 | 0.479 | 0.550 | 1.000 |  |  |  |  |  |  |  |  |  |  |
| I14 | 0.295 | 0.437 | 0.292 | 0.411 | 0.546 | 0.532 | 0.256 | 0.594 | 0.614 | 0.531 | 0.520 | 0.549 | 1.000 |  |  |  |  |  |  |  |  |  |
| I15 | 0.212 | 0.294 | 0.244 | 0.247 | 0.429 | 0.434 | 0.250 | 0.412 | 0.459 | 0.444 | 0.510 | 0.548 | 0.505 | 1.000 |  |  |  |  |  |  |  |  |
| I18 | 0.260 | 0.292 | 0.273 | 0.249 | 0.501 | 0.423 | 0.235 | 0.431 | 0.453 | 0.455 | 0.504 | 0.449 | 0.493 | 0.417 | 1.000 |  |  |  |  |  |  |  |
| I19 | 0.316 | 0.434 | 0.310 | 0.384 | 0.416 | 0.391 | 0.215 | 0.441 | 0.459 | 0.493 | 0.501 | 0.450 | 0.495 | 0.356 | 0.411 | 1.000 |  |  |  |  |  |  |
| I20 | 0.254 | 0.322 | 0.322 | 0.294 | 0.439 | 0.376 | 0.140 | 0.417 | 0.397 | 0.418 | 0.521 | 0.426 | 0.424 | 0.404 | 0.503 | 0.481 | 1.000 |  |  |  |  |  |
| I21 | 0.355 | 0.499 | 0.447 | 0.423 | 0.473 | 0.473 | 0.215 | 0.527 | 0.546 | 0.489 | 0.490 | 0.411 | 0.532 | 0.338 | 0.400 | 0.592 | 0.563 | 1.000 |  |  |  |  |
| I22 | 0.349 | 0.463 | 0.341 | 0.382 | 0.535 | 0.554 | 0.278 | 0.585 | 0.592 | 0.571 | 0.584 | 0.576 | 0.627 | 0.476 | 0.556 | 0.583 | 0.532 | 0.620 | 1.000 |  |  |  |
| I23 | 0.272 | 0.414 | 0.328 | 0.343 | 0.428 | 0.408 | 0.203 | 0.453 | 0.482 | 0.465 | 0.454 | 0.392 | 0.511 | 0.361 | 0.389 | 0.563 | 0.428 | 0.524 | 0.587 | 1.000 |  |  |
| I24 | 0.208 | 0.243 | 0.198 | 0.191 | 0.332 | 0.361 | 0.189 | 0.344 | 0.436 | 0.358 | 0.403 | 0.372 | 0.343 | 0.377 | 0.456 | 0.248 | 0.375 | 0.320 | 0.413 | 0.330 | 1.000 |  |
| I26 | 0.329 | 0.404 | 0.275 | 0.350 | 0.553 | 0.607 | 0.294 | 0.562 | 0.561 | 0.528 | 0.503 | 0.484 | 0.573 | 0.442 | 0.547 | 0.486 | 0.407 | 0.536 | 0.633 | 0.481 | 0.452 | 1.000 |

Table 3. Correlation matrix of exploratory factor analysis; all p (1-tailed) < .05.

| Numbers of item | I1 | I2 | I3 | I4 | I5 | I7 | I8 | I9 | I10 | I11 | I12 | I13 | I14 | I15 | I18 | I19 | I20 | I21 | I22 | I23 | I24 | I26 |
| --- | --- | --- | --- | --- | --- | --- | --- | --- | --- | --- | --- | --- | --- | --- | --- | --- | --- | --- | --- | --- | --- | --- |
| I1 | 1.000 |  |  |  |  |  |  |  |  |  |  |  |  |  |  |  |  |  |  |  |  |  |
| I2 | 0.444 | 1.000 |  |  |  |  |  |  |  |  |  |  |  |  |  |  |  |  |  |  |  |  |
| I3 | 0.360 | 0.306 | 1.000 |  |  |  |  |  |  |  |  |  |  |  |  |  |  |  |  |  |  |  |
| I4 | 0.381 | 0.330 | 0.228 | 1.000 |  |  |  |  |  |  |  |  |  |  |  |  |  |  |  |  |  |  |
| I5 | 0.362 | 0.326 | 0.214 | 0.386 | 1.000 |  |  |  |  |  |  |  |  |  |  |  |  |  |  |  |  |  |
| I7 | 0.395 | 0.337 | 0.286 | 0.433 | 0.499 | 1.000 |  |  |  |  |  |  |  |  |  |  |  |  |  |  |  |  |
| I8 | 0.370 | 0.360 | 0.412 | 0.254 | 0.472 | 0.623 | 1.000 |  |  |  |  |  |  |  |  |  |  |  |  |  |  |  |
| I9 | 0.424 | 0.456 | 0.339 | 0.440 | 0.472 | 0.679 | 0.567 | 1.000 |  |  |  |  |  |  |  |  |  |  |  |  |  |  |
| I10 | 0.395 | 0.441 | 0.347 | 0.261 | 0.592 | 0.565 | 0.731 | 0.535 | 1.000 |  |  |  |  |  |  |  |  |  |  |  |  |  |
| I11 | 0.441 | 0.429 | 0.350 | 0.430 | 0.491 | 0.570 | 0.615 | 0.688 | 0.669 | 1.000 |  |  |  |  |  |  |  |  |  |  |  |  |
| I12 | 0.377 | 0.419 | 0.294 | 0.411 | 0.533 | 0.510 | 0.656 | 0.581 | 0.663 | 0.622 | 1.000 |  |  |  |  |  |  |  |  |  |  |  |
| I13 | 0.264 | 0.279 | 0.243 | 0.293 | 0.372 | 0.513 | 0.339 | 0.420 | 0.351 | 0.452 | 0.385 | 1.000 |  |  |  |  |  |  |  |  |  |  |
| I14 | 0.288 | 0.327 | 0.219 | 0.384 | 0.399 | 0.599 | 0.619 | 0.530 | 0.517 | 0.516 | 0.535 | 0.462 | 1.000 |  |  |  |  |  |  |  |  |  |
| I15 | 0.392 | 0.437 | 0.343 | 0.347 | 0.527 | 0.562 | 0.671 | 0.559 | 0.671 | 0.641 | 0.587 | 0.465 | 0.523 | 1.000 |  |  |  |  |  |  |  |  |
| I18 | 0.344 | 0.352 | 0.250 | 0.309 | 0.259 | 0.380 | 0.394 | 0.490 | 0.348 | 0.483 | 0.372 | 0.448 | 0.460 | 0.487 | 1.000 |  |  |  |  |  |  |  |
| I19 | 0.250 | 0.337 | 0.329 | 0.098 | 0.334 | 0.320 | 0.399 | 0.361 | 0.422 | 0.418 | 0.393 | 0.435 | 0.456 | 0.395 | 0.486 | 1.000 |  |  |  |  |  |  |
| I20 | 0.291 | 0.392 | 0.319 | 0.293 | 0.367 | 0.536 | 0.483 | 0.582 | 0.512 | 0.518 | 0.502 | 0.383 | 0.511 | 0.495 | 0.402 | 0.470 | 1.000 |  |  |  |  |  |
| I21 | 0.222 | 0.097 | 0.300 | 0.217 | 0.366 | 0.440 | 0.359 | 0.226 | 0.276 | 0.317 | 0.219 | 0.429 | 0.452 | 0.423 | 0.220 | 0.434 | 0.349 | 1.000 |  |  |  |  |
| I22 | 0.476 | 0.478 | 0.321 | 0.442 | 0.500 | 0.570 | 0.551 | 0.553 | 0.472 | 0.497 | 0.574 | 0.511 | 0.532 | 0.613 | 0.398 | 0.412 | 0.519 | 0.514 | 1.000 |  |  |  |
| I23 | 0.372 | 0.444 | 0.351 | 0.426 | 0.525 | 0.556 | 0.580 | 0.575 | 0.568 | 0.611 | 0.621 | 0.412 | 0.542 | 0.665 | 0.591 | 0.462 | 0.540 | 0.357 | 0.564 | 1.000 |  |  |
| I24 | 0.304 | 0.270 | 0.243 | 0.229 | 0.453 | 0.484 | 0.530 | 0.404 | 0.515 | 0.478 | 0.631 | 0.364 | 0.455 | 0.471 | 0.310 | 0.395 | 0.591 | 0.410 | 0.538 | 0.530 | 1.000 |  |
| I26 | 0.195 | 0.238 | 0.212 | -0.015 | 0.081 | 0.168 | 0.330 | 0.279 | 0.265 | 0.297 | 0.254 | 0.156 | 0.321 | 0.260 | 0.308 | 0.440 | 0.208 | 0.236 | 0.197 | 0.308 | 0.248 | 1.000 |

Table 4. Correlation matrix of confirmatory factor analysis; all p (1-tailed) < .05

| Total variance explained of exploratory factor analysis | | | | | | | | | |
| --- | --- | --- | --- | --- | --- | --- | --- | --- | --- |
| Components | Initial eigenvalue | | | Extraction sums of squared loadings | | | Rotation sums of squared loadings | | |
|  | Total | % of Variance | Cumulative % | Total | % of Variance | Cumulative % | Total | % of Variance | Cumulative % |
| 1 | 9.917 | 45.080 | 45.080 | 9.917 | 45.080 | 45.080 | 6.855 | 31.157 | 31.157 |
| 2 | 1.318 | 5.989 | 51.069 | 1.318 | 5.989 | 51.069 | 4.381 | 19.912 | 51.069 |
| 3 | 0.995 | 4.525 | 55.593 |  | | | | | |
| 4 | 0.940 | 4.271 | 59.864 |  |  |  |  |  |  |
| 5 | 0.835 | 3.796 | 63.661 |  |  |  |  |  |  |
| 6 | 0.766 | 3.483 | 67.143 |  |  |  |  |  |  |
| 7 | 0.744 | 3.383 | 70.526 |  |  |  |  |  |  |
| 8 | 0.672 | 3.053 | 73.580 |  |  |  |  |  |  |
| 9 | 0.629 | 2.860 | 76.439 |  |  |  |  |  |  |
| 10 | 0.572 | 2.599 | 79.039 |  |  |  |  |  |  |
| 11 | 0.547 | 2.486 | 81.525 |  |  |  |  |  |  |
| 12 | 0.498 | 2.264 | 83.789 |  |  |  |  |  |  |
| 13 | 0.486 | 2.208 | 85.996 |  |  |  |  |  |  |
| 14 | 0.438 | 1.993 | 87.989 |  |  |  |  |  |  |
| 15 | 0.410 | 1.865 | 89.855 |  |  |  |  |  |  |
| 16 | 0.388 | 1.765 | 91.619 |  |  |  |  |  |  |
| 17 | 0.369 | 1.676 | 93.295 |  |  |  |  |  |  |
| 18 | 0.331 | 1.506 | 94.801 |  |  |  |  |  |  |
| 19 | 0.311 | 1.413 | 96.214 |  |  |  |  |  |  |
| 20 | 0.298 | 1.356 | 97.570 |  |  |  |  |  |  |
| 21 | 0.280 | 1.271 | 98.841 |  |  |  |  |  |  |
| 22 | 0.255 | 1.159 | 100.000 |  |  |  |  |  |  |
| Extraction Method: Principal Component Analysis | | | | | | | | | |

Table 5. Total variance explained of exploratory factor analysis

| Total variance explained of confirmatory factor analysis | | | | | | | | | |
| --- | --- | --- | --- | --- | --- | --- | --- | --- | --- |
| Components | Initial eigenvalue | | | Extraction sums of squared loadings | | | Rotation sums of squared loadings | | |
|  | Total | % of Variance | Cumulative % | Total | % of Variance | Cumulative % | Total | % of Variance | Cumulative % |
| 1 | 10.799 | 44.997 | 44.997 | 10.799 | 44.997 | 44.997 | 7.732 | 32.217 | 32.217 |
| 2 | 1.420 | 5.915 | 50.912 | 1.420 | 5.915 | 50.912 | 4.487 | 18.695 | 50.912 |
| 3 | 1.334 | 5.557 | 56.469 |  | | | | | |
| 4 | 1.146 | 4.775 | 61.244 |  |  |  |  |  |  |
| 5 | 1.044 | 4.349 | 65.593 |  |  |  |  |  |  |
| 6 | 0.873 | 3.637 | 69.230 |  |  |  |  |  |  |
| 7 | 0.721 | 3.004 | 72.234 |  |  |  |  |  |  |
| 8 | 0.706 | 2.940 | 75.174 |  |  |  |  |  |  |
| 9 | 0.671 | 2.794 | 77.968 |  |  |  |  |  |  |
| 10 | 0.602 | 2.510 | 80.479 |  |  |  |  |  |  |
| 11 | 0.569 | 2.372 | 82.851 |  |  |  |  |  |  |
| 12 | 0.545 | 2.270 | 85.121 |  |  |  |  |  |  |
| 13 | 0.495 | 2.062 | 87.183 |  |  |  |  |  |  |
| 14 | 0.473 | 1.972 | 89.155 |  |  |  |  |  |  |
| 15 | 0.404 | 1.682 | 90.838 |  |  |  |  |  |  |
| 16 | 0.363 | 1.511 | 92.349 |  |  |  |  |  |  |
| 17 | 0.321 | 1.338 | 93.687 |  |  |  |  |  |  |
| 18 | 0.288 | 1.201 | 94.888 |  |  |  |  |  |  |
| 19 | 0.271 | 1.130 | 96.017 |  |  |  |  |  |  |
| 20 | 0.236 | 0.983 | 97.001 |  |  |  |  |  |  |
| 21 | 0.215 | 0.896 | 97.001 |  |  |  |  |  |  |
| 22 | 0.197 | 0.822 | 98.719 |  |  |  |  |  |  |
| 23 | 0.167 | 0.695 | 99.414 |  | | | | | |
| 24 | 0.141 | 0.586 | 100.000 |  | | | | | |
| Extraction Method: Principal Component Analysis | | | | | | | | | |

Table 6. Total variance explained of confirmatory factor analysis
