# Supplementary material for: A novel instrument of cognitive and social congruence within peer-assisted learning in medical training: construction of a questionnaire by factor analyses
Source: BMC Med Educ. 2020 Jul 8;20:214. doi: 10.1186/s12909-020-02129-x (PMC7346370; doi:10.1186/s12909-020-02129-x)
Supplement: Supplementary file 2 — Additional file 2: Figure 2. Screeplot exploratory and confirmatory factor analysis. Both screeplots presented a two-factor solution: For students (exploratory factor analysis), the scree plot and the Kaiser Criterion indicated a two-factor solution with Eigenvalue (1) = 9.92 and Eigenvalue (2) = 1.32. For student tutors (confirmatory factor analysis), the results of the Kaiser Criterion and screeplot strengthened the two-factor solution with Eigenvalue (1) = 10.80 and Eigenvalue (2) = 1.42. [file 12909_2020_2129_MOESM2_ESM.docx]

Figure 2. Screeplot of exploratory and confirmatory factor analysis
